# Supplementary material for: Evolution of sequence-specific anti-silencing systems in Arabidopsis
Source: Nat Commun. 2017 Dec 18;8:2161. doi: 10.1038/s41467-017-02150-7 (PMC5735166; doi:10.1038/s41467-017-02150-7)
Supplement: Supplementary file 1 — Supplementary Information [file 41467_2017_2150_MOESM1_ESM.pdf]

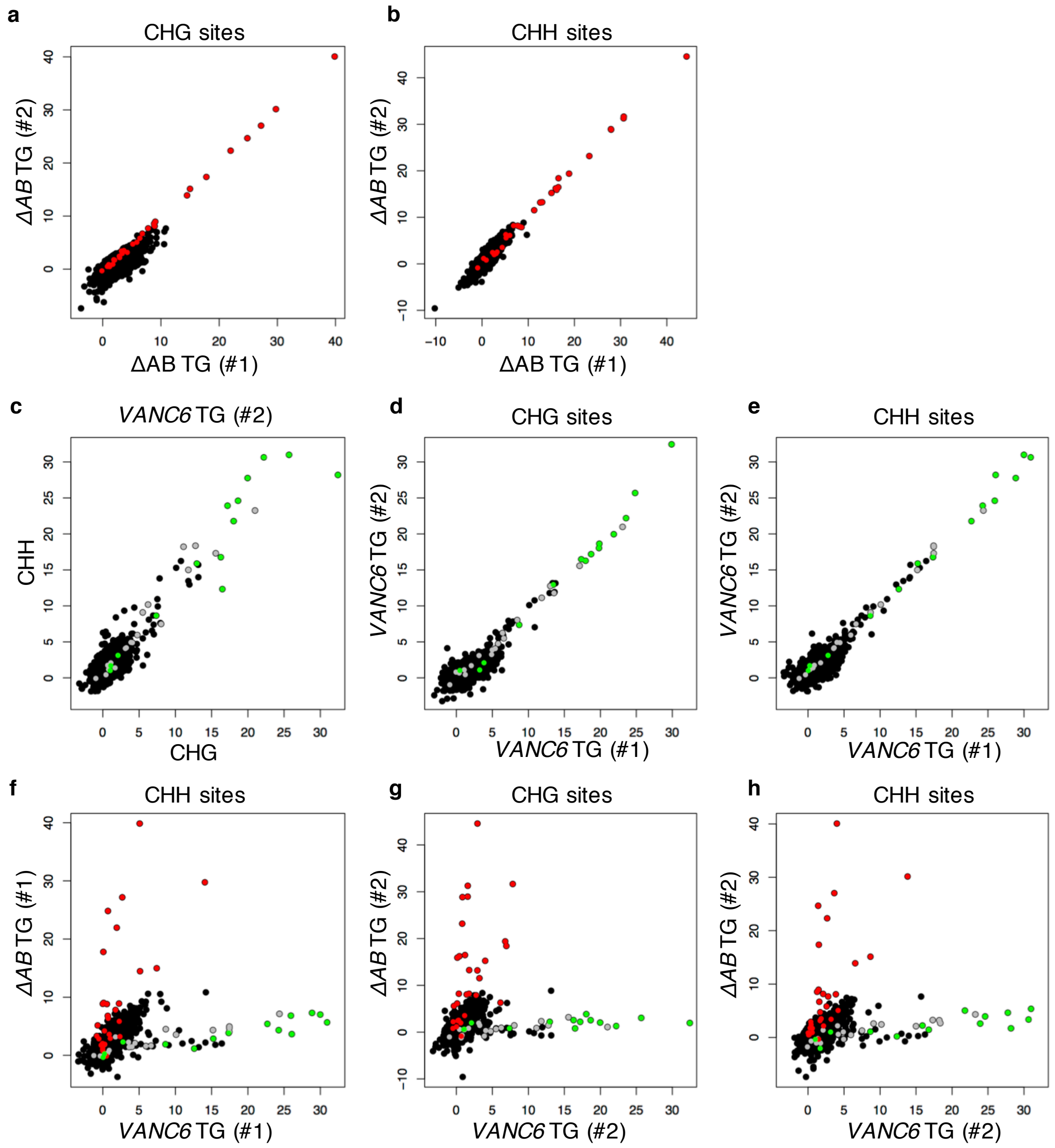

Supplementary Figure 1 I Effects of VANC transgenes: extension of results in Figure 1. a, b, Scatter plots comparing effects on DNA methylation in TEs between two biological replicates (#1 and #2) of *ΔAB* transgenic plants on CHG sites (a) and CHH sites (b). **c,** Decreases in TE DNA methylation of another *VANC6* transgenic line (#2) at CHG sites and CHH sites. **d, e,** Scatter plots comparing effects on DNA methylation in TEs between two biological replicates (#1 and #2) of *VANC6* transgenic plants on CHG sites (d) and CHH sites (e). **f-h,** Comparison of decreases in TE DNA methylation between *ΔAB* and *VANC6* transgenic plants.

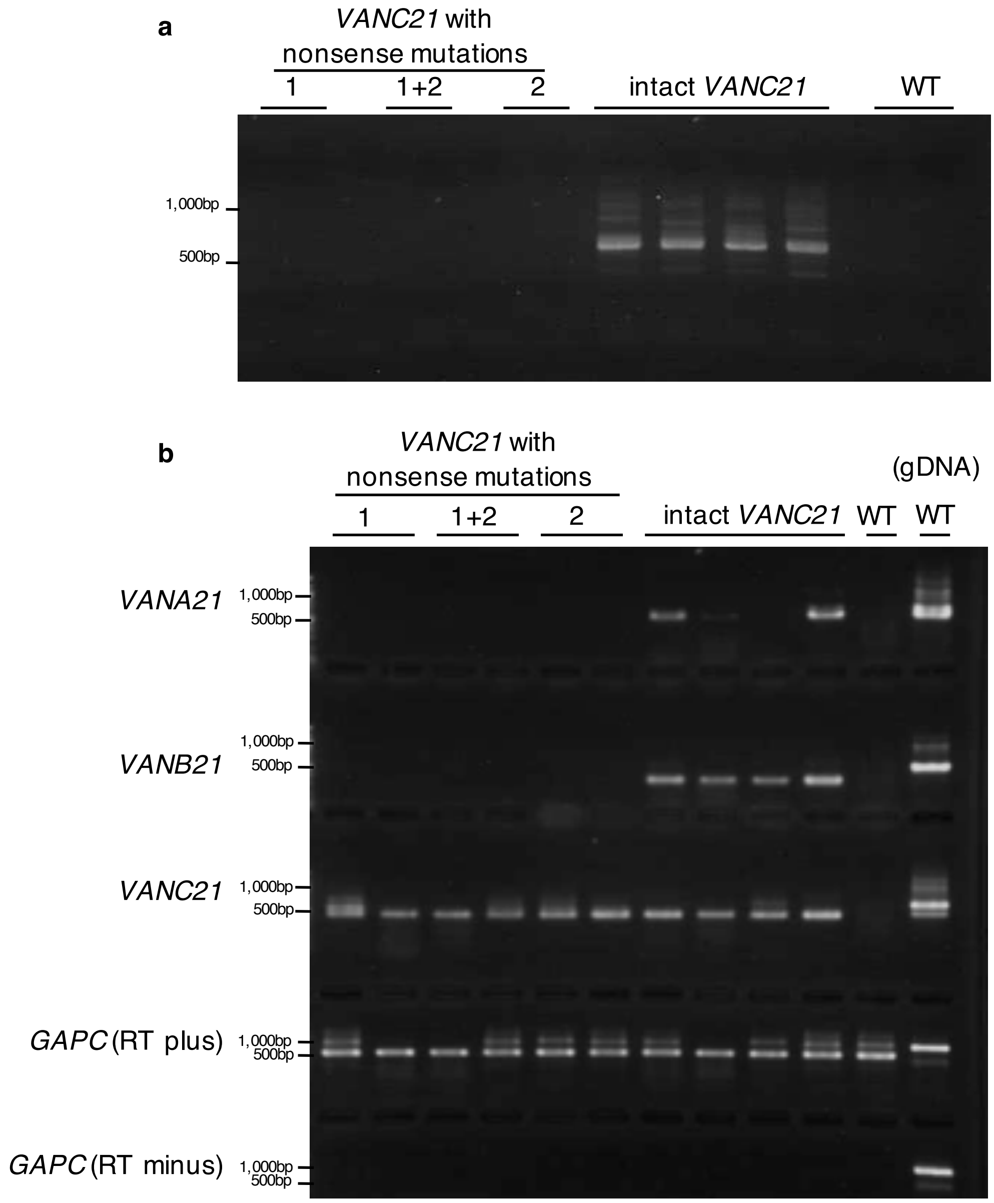

Supplementary Figure 2 I Effects of nonsense mutations of *VANC21* transgene on excision of endogenous *Hi* and transcription of genes encoded in endogenous *Hi*. Conditions for detecting the excision and transcription are as described previously[18]. **a,** Excision of endogenous *Hi* could not be detected by *VANC21* transgenes with nonsense mutations 1, 2, or both (1+2). Four transgenic lines with intact *VANC21* are shown as positive controls. WT: Wild type non-transgenic plants. **b,** Expression of genes encoded in endogenous *Hi*. Expression could not be detected for *VANA21* and *VANB21* in *VANC21* transgenic lines with nonsense mutations 1, 2, or both (1+2).

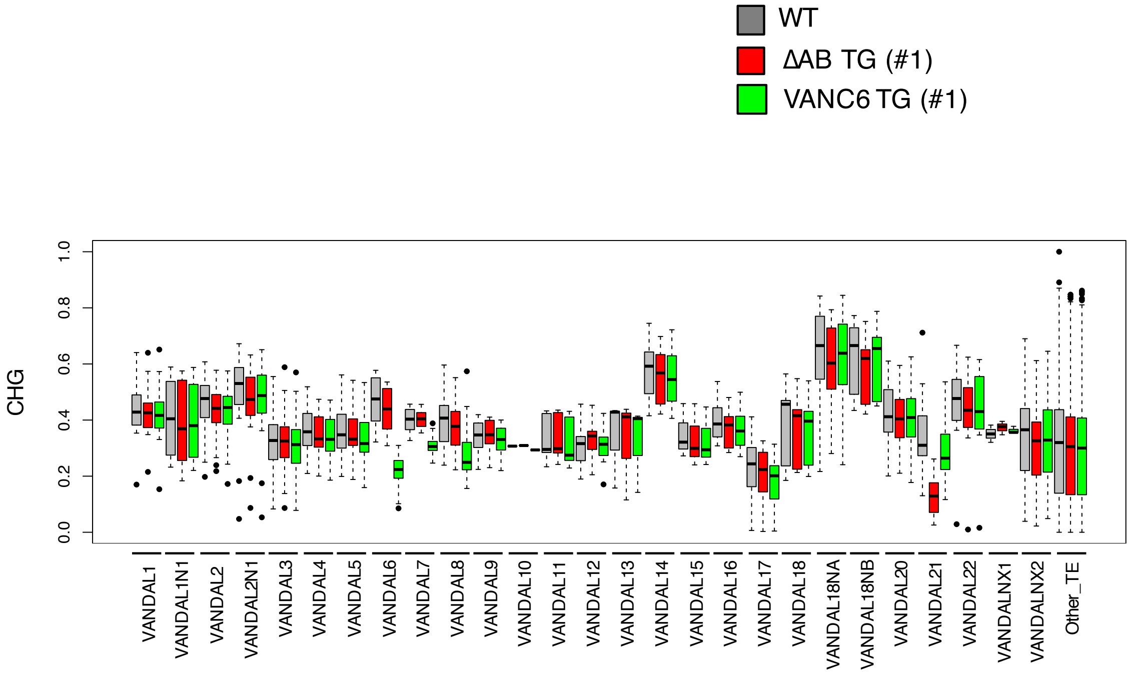

Supplementary Figure 3 l Effects of VANC transgenes. Boxplots show DNA methylation levels of TEs at CHG sites for each of *VANDAL* families. TEs longer than 1 kb are used. DNA methylation levels are assessed by dividing methylated cytosine counts by total cytosine counts mapped for each TE. Original DNA methylation data for TEs longer than 1 kb are in Supplementary Data 1.

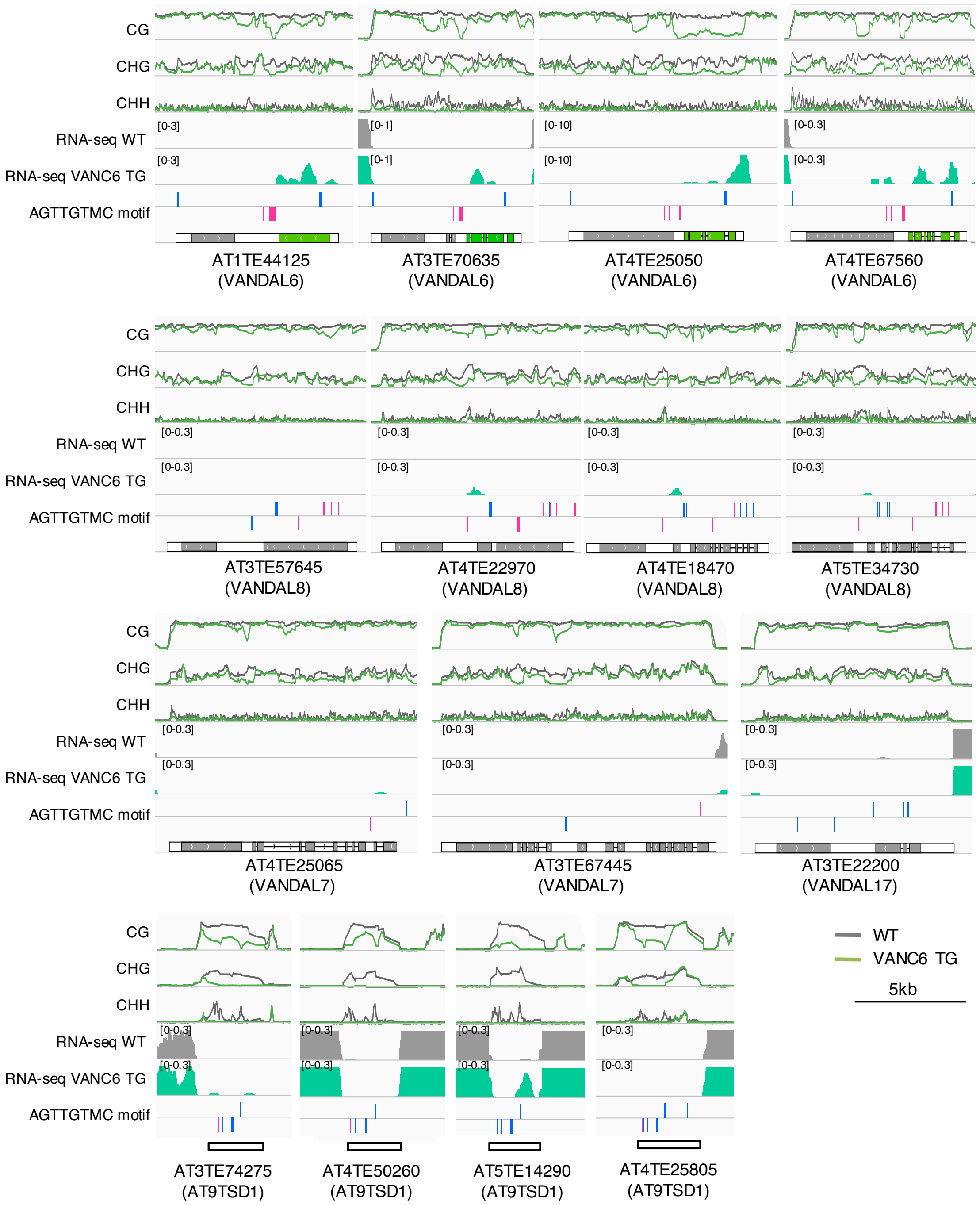

Supplementary Figure 4 I Integrative genome views at VANC6 DMRs. Genome browser views showing the DNA methylation (0-100%) and RPM (reads per million mapped reads)-normalized RNA profiles of WT and VANC6 transgenic plants at TEs with VANC6 DMRs. *VANC6* exons are colored green.

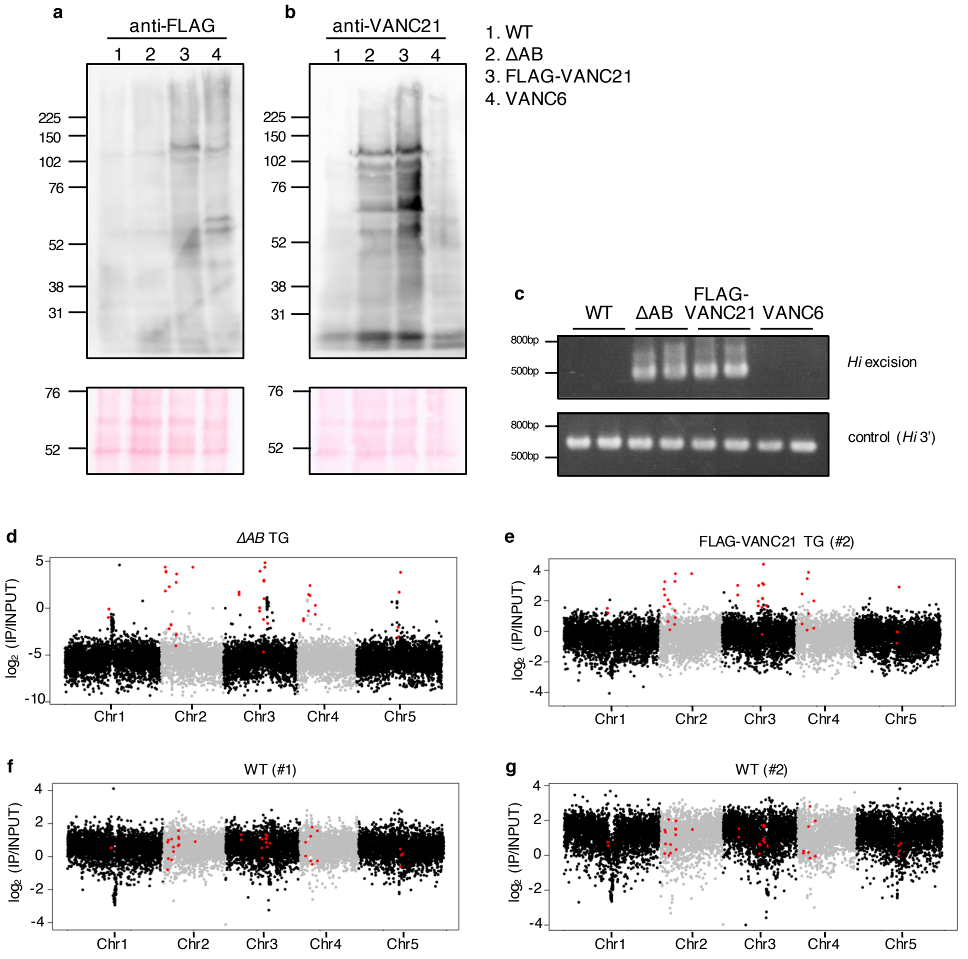

Supplementary Figure 5 I ChIP-seq analyses using anti-FLAG antibody and anti-VANC21 antibody. a, b, Western blotting of nuclear extracts using anti-FLAG antibody (a) and anti-VANC21 antibody (b). Wild type plants, *ΔAB* transgenic plants, *FLAG-VANC21* transgenic plants, and *VANC6* transgenic plants were used for lanes 1-4, respectively. Lower panels indicate loading controls. **c,** Excision of endogenous *Hi* detected in transgenic plants expressing *FLAG-VANC21*. *ΔAB* and *VANC6* transgenic plants are shown as positive and negative controls, respectively. *Hi* 3' region was examined as a control. **d,** ChIP-seq results using anti-VANC21 polyclonal antibody for *ΔAB* transgenic plants. **e-g,** Two biological replicates of ChIP-seq results using anti-FLAG antibody for plants with the tagged transgene (e) and control non-transgenic plants (WT) plants (f, g) examined in parallel. Red dots indicate the regions including *VANDAL21* copies more than 1kb long. The regions with satellite repeats are excluded from the plots.

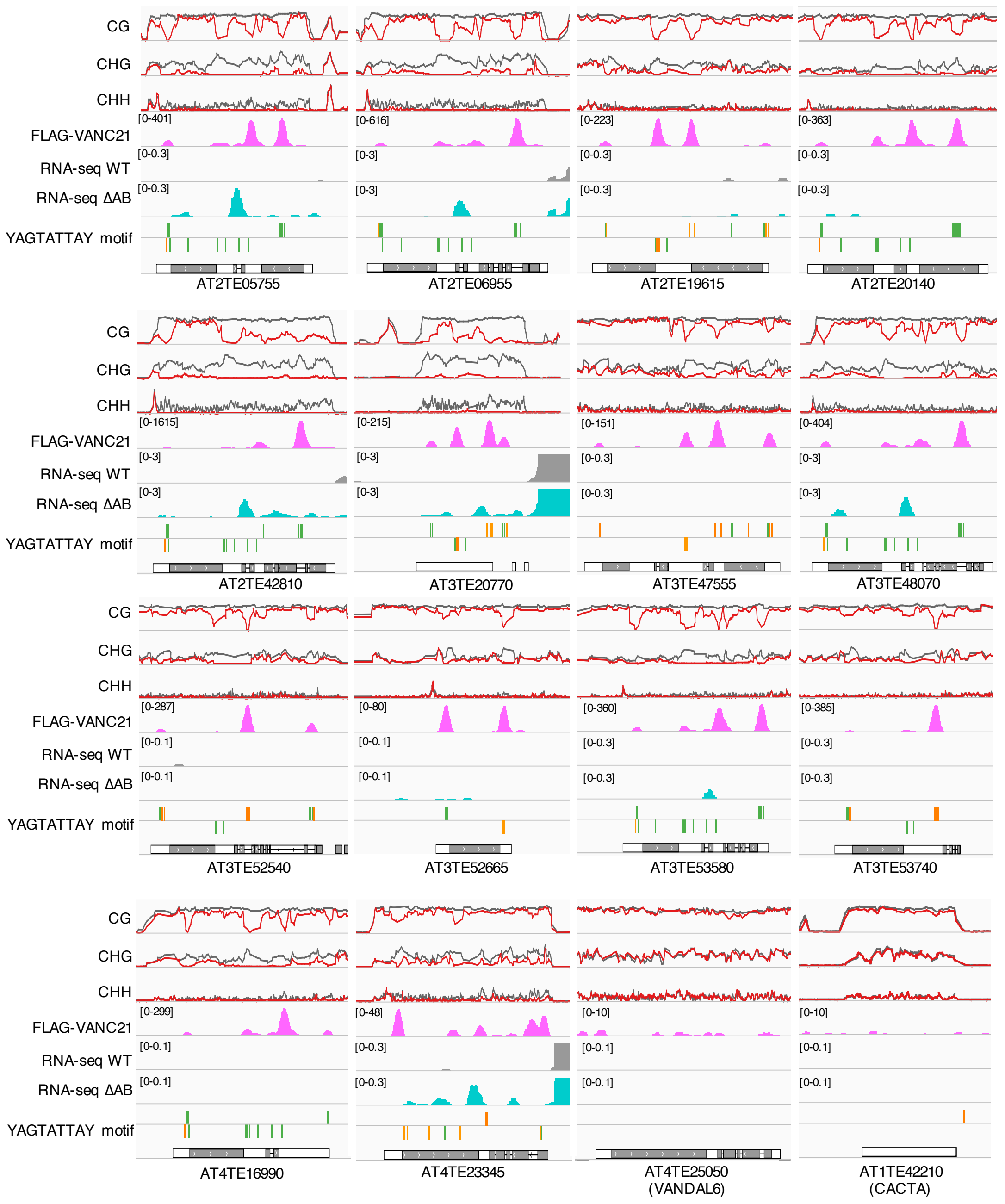

Supplementary Figure 6 I Integrative genome views at VANC21 binding regions. The formats are as shown in Figure 2b, with adding RPM (reads per million mapped reads)-normalized RNA profiling of WT (gray) and *ΔAB* plants (light blue).

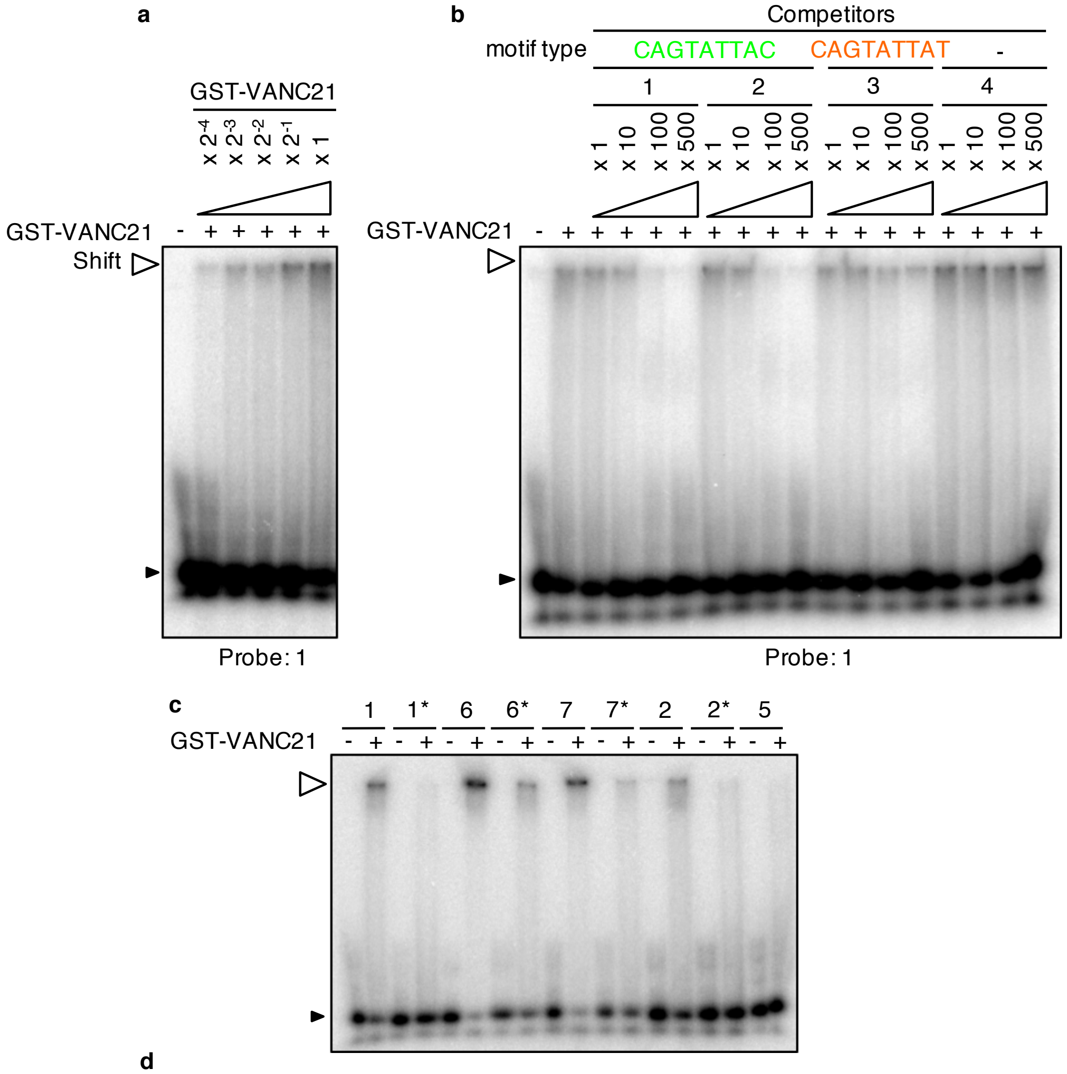

d

1	AATGTTTAAATAGCAC**CAGTATTAC**TTAGACACGTAGTAT	AT2TE42810 VANA21 upstream
1*	AATGTTTAAATAGCAC**CAGCATTAC**TTAGACACGTAGTAT	
2	AATTCCCCGTAATTAC**CAGTATTAC**ACAGATACTCCTATT	AT2TE42810 VANC21 intron
2*	AATTCCCCGTAATTAC**CAGCATTAC**ACAGATACTCCTATT	
3	ATTTCCCTTGTATTCT**CAGTATTAT**CGTTAAATCCTAGTA	AT3TE52540 VANB21 intron
3*	ATTTCCCTTGTATTCT**CAGCATTAT**CGTTAAATCCTAGTA	
4	AGATACTTCGGAATTGGTTTTCATGACGGACAGAAATGCA	AT2TE42810 VANA21_exon
5	AACGGCACAAACGACTGCGACGGCGATGGCAACGACAGCA	AT2TE42810 VANC21_exon
6	ATCCATAACACCAACT**TAGTATTAC**CAGTAGTACTAATAT	AT2TE42810 VANA21-VANB21 intergenic
6*	ATCCATAACACCAACT**TAGCATTAC**CAGTAGTACTAATAT	
7	AAATTTAGTTAAACCT**TAGTATTAC**GCGATATTACCAATT	AT2TE42810 VANB21-VANC21 intergenic
7*	AAATTTAGTTAAACCT**TAGCATTAC**GCGATATTACCAATT	

Supplementary Figure 7 I EMSA by VANC21 protein. a, EMSA with 2-fold serial dilutions of GST-VANC21 protein samples. **b,** Competitor assay with unlabeled dsDNA sequences. dsDNAs containing the CAGTATTAC motif (DNA 1 and 2) effectively outcompete interaction between GST-VANC21 and labeled dsDNA. **c,** EMSA assay with additional probes. **d,** Sequences of the dsDNA probe used in EMSA (a-c).

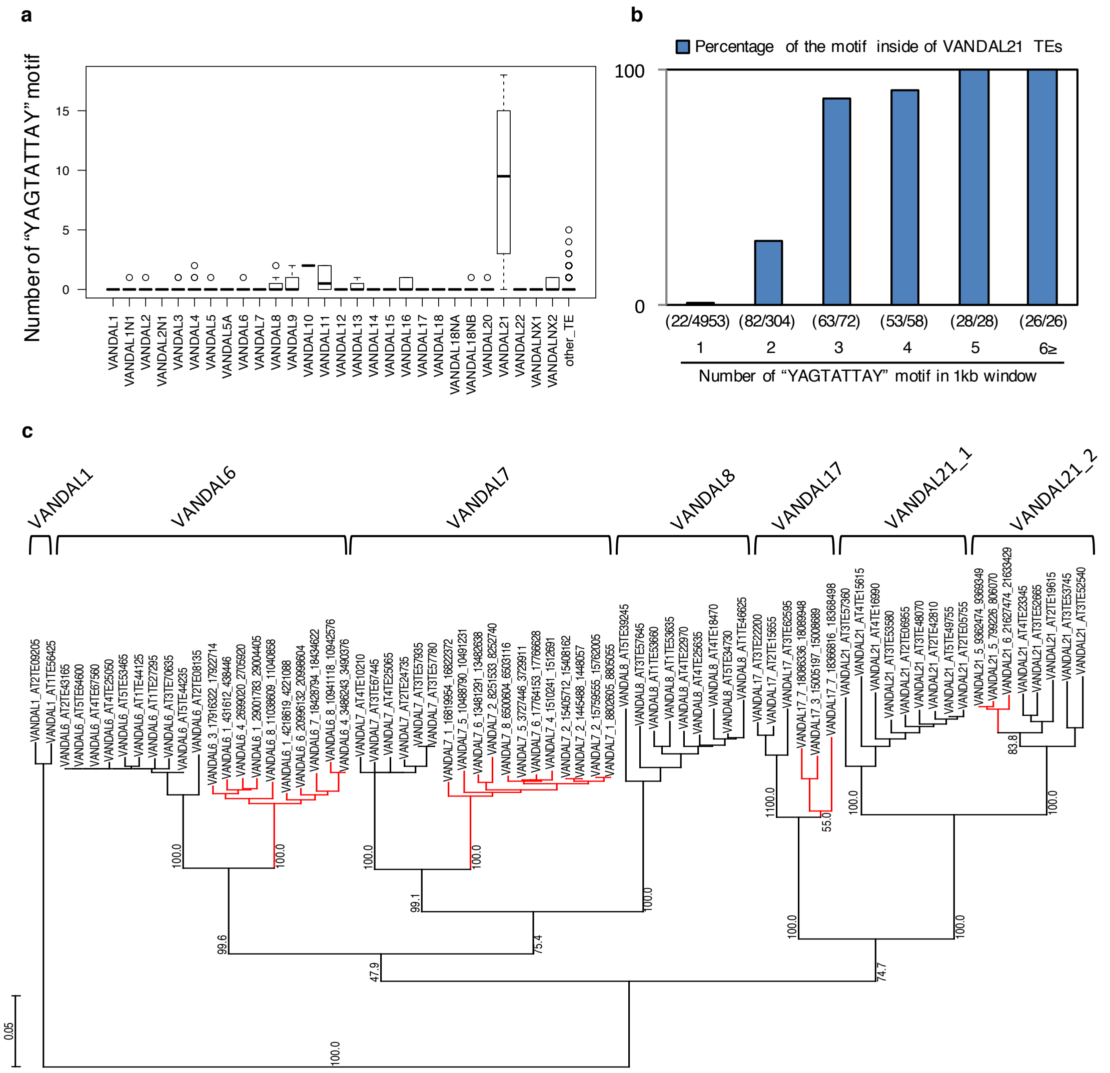

Supplementary Figure 8 l Distribution of VANC21 recognition motifs. a, Number of "YAGTATTAY" motif in VANDAL family members. TEs longer than 1kb were characterized. **b,** Proportion of the YAGTATTAY sites within *VANDAL21* copies after separating the sites by the motif density (motif number per 1kb). Numbers of motifs in total and in *VANDAl21* are also shown. **c,** The phylogenic tree of Figure 3, with adding IDs (TAIR10) for *A. thaliana* TEs, and chromosome numbers and regions for *A. lyrata* TEs. The bootstrap probabilities (%) with 1000 replications for major clusters are indicated beside the branches.

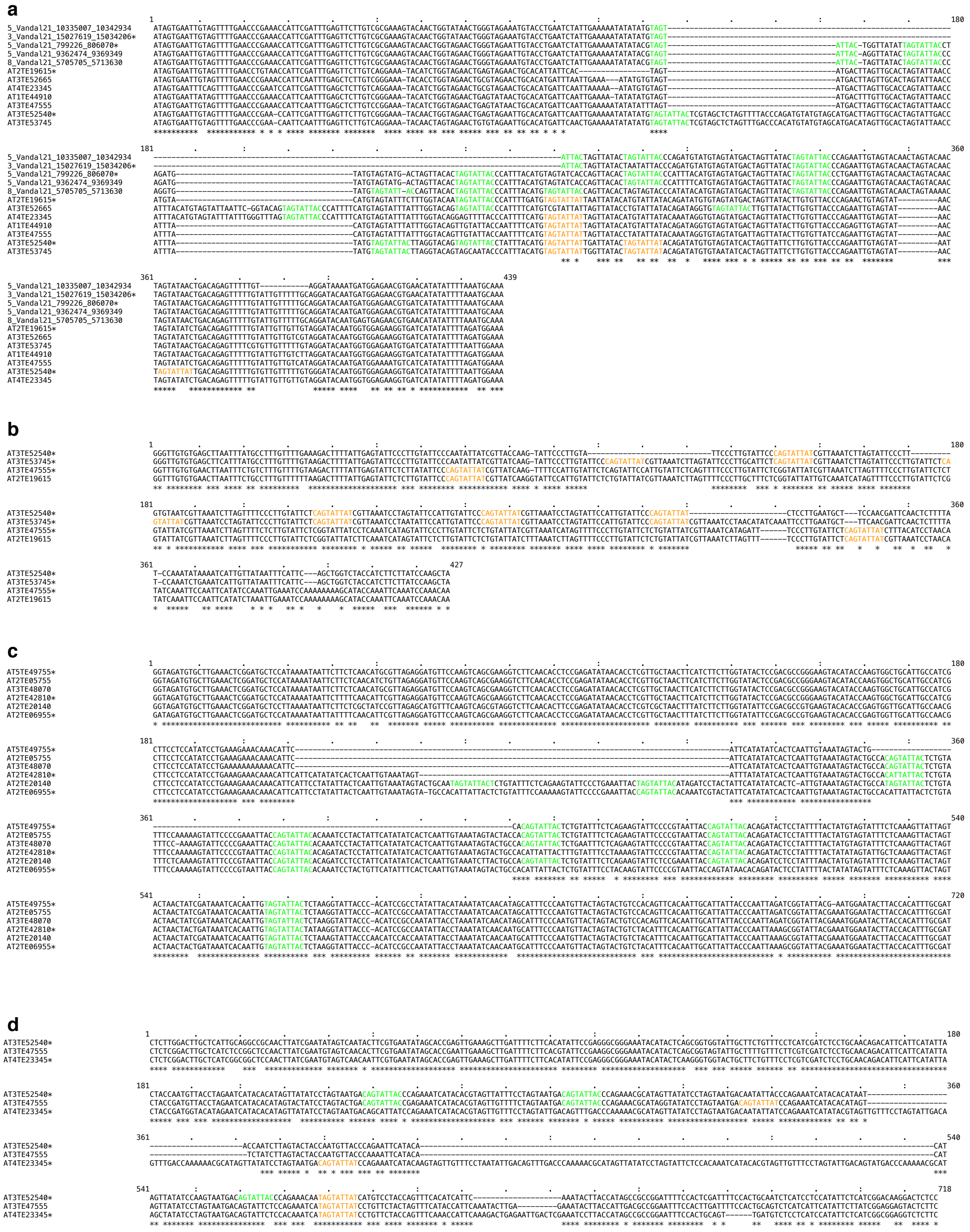

Supplementary Figure 9 | Alignments of tandem repeat regions within *VANDAL21* copies. Alignment of sequences of *VANDAL21* copies in upstream regions of *VANA21* (**a**), intronic regions of *VANB21* (**b**), and intronic regions of *VANC21* (**c, d**). TE names with asterisks are those used for dot-plot in Fig. 4.

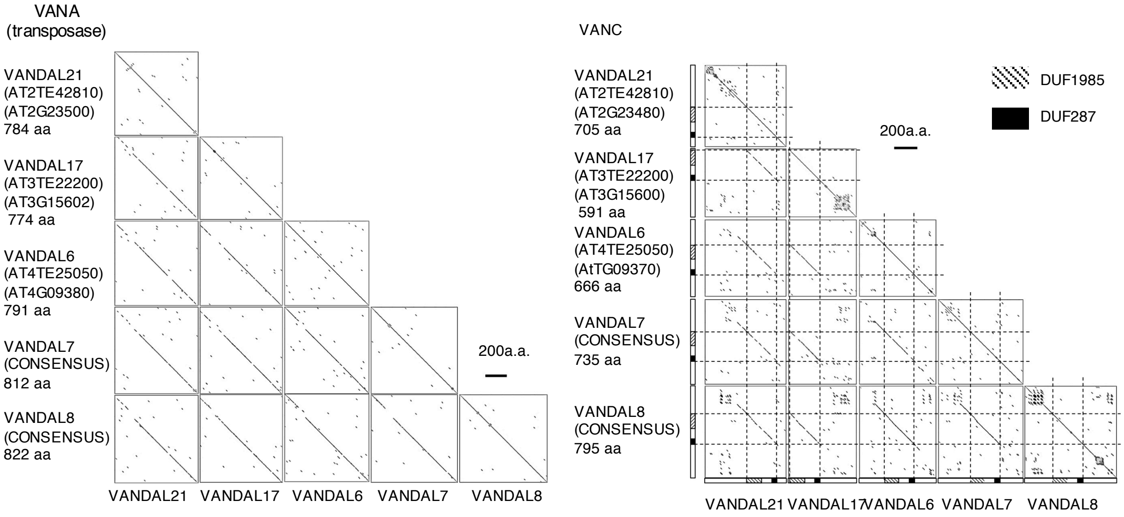

Supplementary Figure 10 l Dot-plot analyses of the transposases (VANA) and anti-silencing proteins (VANC). Comparison on amino-acid sequences of the VANA and VANC proteins. Homologous regions were plotted with dotmatcher program (window size: 10, threshold: 23). Amino acid sequences (N‘ to C’) were ordered from top to bottom and left to right. Figures were made in the same scale. Scale bar for 200 a.a. was shown in the right of plots. For VANC, two domains (DUF1985 and DUF287) were shown by shaded and filled areas, respectively.

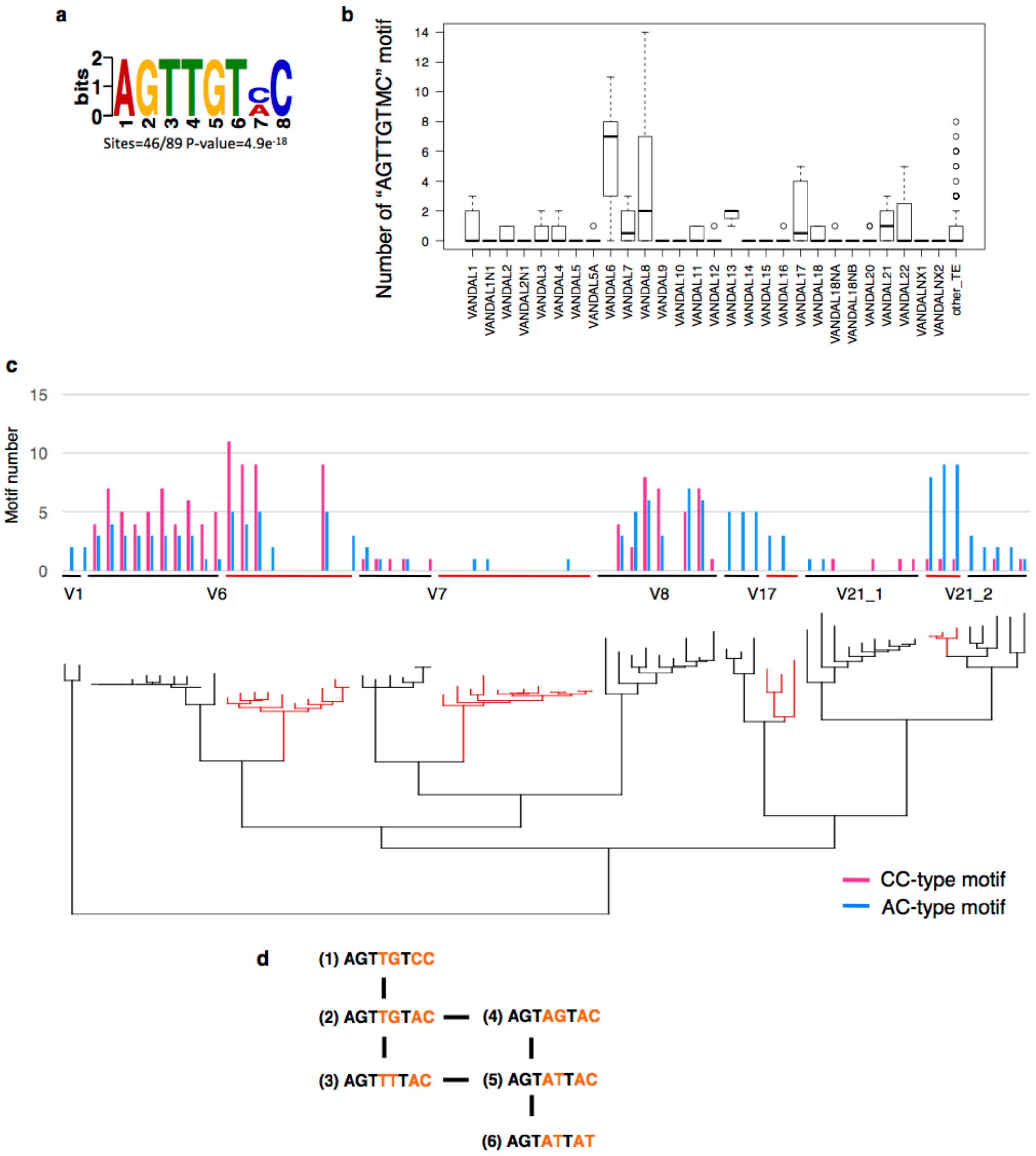

Supplementary Figure 11 I Distribution of VANC6 recognition motifs. a, DNA sequence motif most commonly found at CG-DMRs in VANC6 transgenic plants. Localizations of the motifs in each of the TEs are shown in Supplementary Fig. 4. **b,** Number of "AGTTGTMC" (M=A or C) motif in VANDAL family members. TEs longer than 1kb were characterized. **c,** Numbers of CC- and AC-type motifs within *VANDAL21* and related *VANDAL* family members within the genomes of *A. thaliana* and *A. lyrata* as in Fig. 3d. **d,** Motifs recognized by VANC6 (1, 2) are at least two nucleotides different from motifs recognized by VANC21 (5, 6). Nucleotides conserved in all the motifs are shown by black and variable nucleotides are shown by orange.

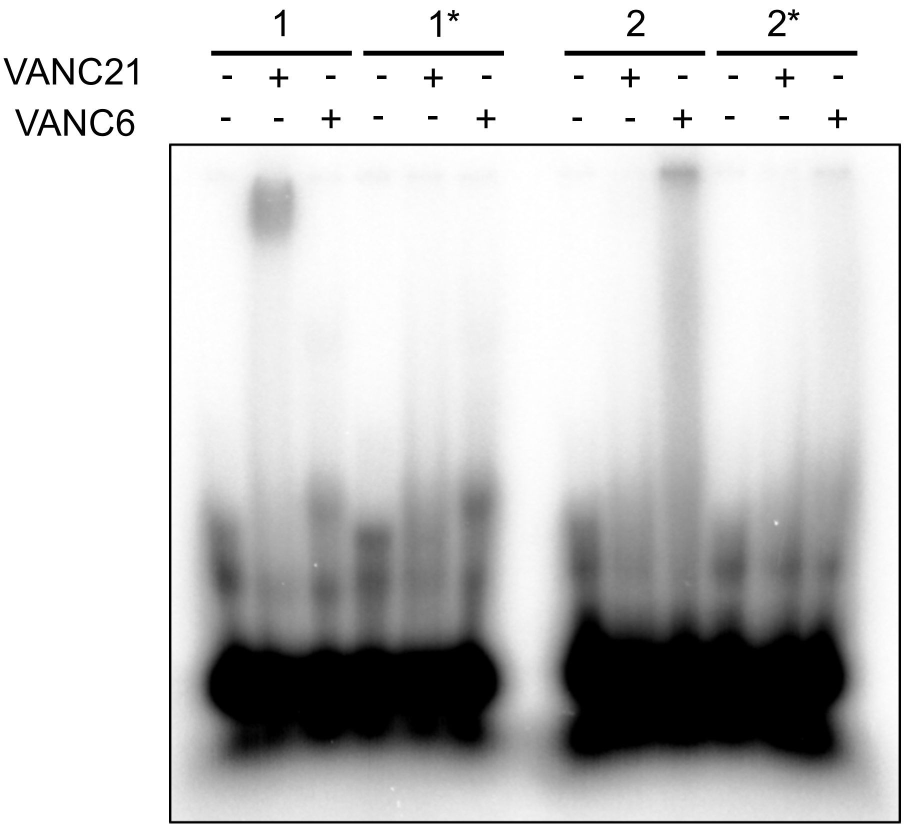

1	AATTACTCGTTCTAGGC**AGTATTAC**CAAAACGGGAAATA	AT2TE42810 VANB21 intron
1*	AATTACTCGTTCTAGGC**AGCATTAC**CAAAACGGGAAATA	
2	GTAGCTA**AGTTGTCC**C**AGTTGGC**CGGTCGATACACATAA	AT4TE25050 VANDAL6 intergenic region
2*	GTAGCTA**AGTTGTCC**C**AGCTGGC**CGGTCGATACACATAA	

Supplementary Figure 12 | Differentiation of binding targets for VANC proteins. EMSA with VANC21 and VANC6 proteins. Sequences of the dsDNA probe used in EMSA are listed in the bottom. The probe 2 has one CC-type motif and one related motif (the third motif for DMRs induced by VANC6 in Supplementary Table 2), and the binding signal became weaker when one of the motifs has a single base substitution.

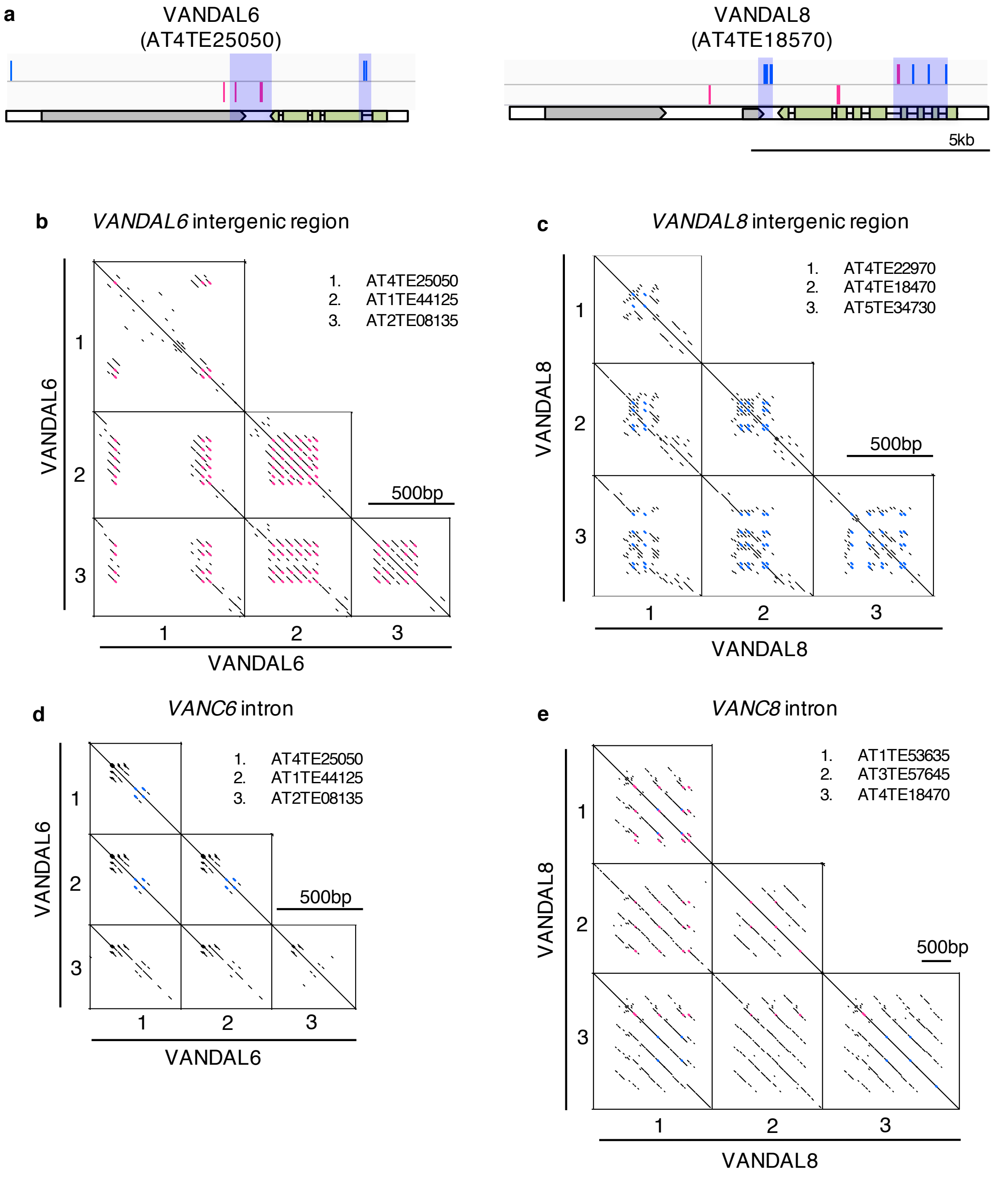

Supplementary Figure 13 l Tandem repeats at DMRs induced by VANC6 TG. a, Structures of *VANDAL6 and VANDAL8* copies, with regions analyzed in (b-e) shown with shadow. Pink and blue bars show CC-type and AC-type motifs, respectively. **b-e,** Dot-plots comparing intergenic regions and introns of VANDAL6 (b, d) and VANDAL8 (c, e). Regions with 10bp exact match are shown by dots as shown in Fig 4. Pink and blue indicate regions with CC-type and AC-type motifs, respectively.

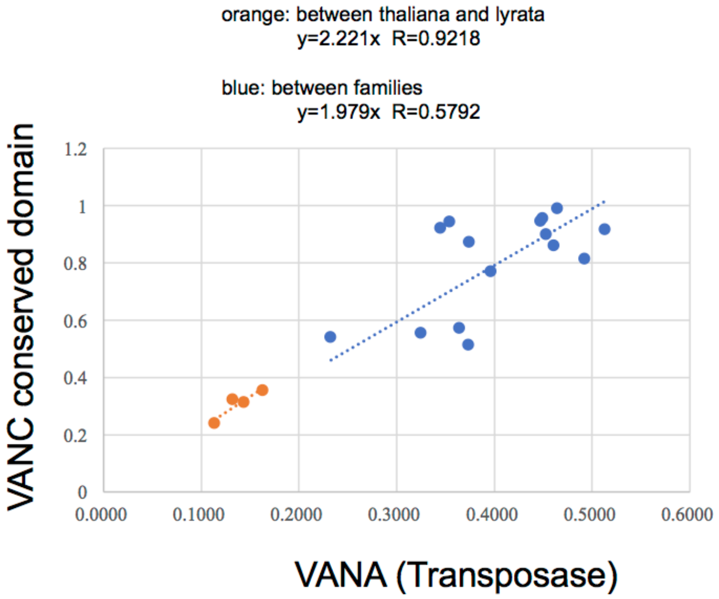

Supplementary Figure 14 | Relationship of poisson distance between VANA and VANC. Blue and orange marks indicate values between *A. thaliana* and *A. lyrata* and between families, respectively. Regression lines and correlation coefficients were estimated with 0 y-intercept.

Supplementary Table 1.

List of overrepresented motifs in VANC21-localized regions (N=89).

Motif[a]	sites[b]	P-value[c]	E-value[d]
YAGTATTAY	69	9.3e-24	2.5e-19
AGTATTYC	36	3.9e-10	9.9e-6
GWAATACC	24	9.9e-9	2.5e-4
AAACAAAS	27	1.2e-8	0.0003
CAATATYA	32	6.6e-8	0.0016
AATCAMAAT	20	2.9e-7	0.007
GTACTMGTA	18	1.5e-6	0.036

a: Overrepresented motif. b: Number of sequences matching the motif. c: The p-value of Fisher’s Exact Test for enrichment of the motif in the sequences. d: The motif p-value times the number of candidate motifs tested.

Supplementary Table 2.
List of overrepresented motifs in CG-DMRs induced by VANC21 (top) or VANC6 (bottom).

CG-DMRs induced by ∆AB at VANDAL21 TEs (N=93).

Motif[a]	sites[b]	P-value[c]	E-value[d]
GTATTHCM	76	4.4e-34	1.2e-29
RRTATTAC	53	2.6e-17	6.9e-13
MTTCGA	40	1.1e-8	0.00029
AYTAGTAC	25	7.1e-8	0.0018
CAGTATTAT	20	3e-7	0.0075

CG-DMRs induced by VANC6 at affected TEs (N=89).

Motif[a]	sites[b]	P-value[c]	E-value[d]
AGTTGTMC	46	4.9e-18	1.2e-13
AARTACCA	31	3.9e-9	9.0e-5
AGTTGGC	23	2.3e-8	5.3e-4
ATACAMATA	25	6.5e-8	1.5e-3
AACATAKT	23	3.3e-7	7.5e-3

Differentially-Methylated Regions at CG-sites (CG-DMRs) induced by VANC genes were defined as previously described[38]. Short motifs were searched in CG-DMRs induced by ∆AB at VANDAL21 TEs (N=93), and in CG-DMRs induced by VANC6 at VANDAL6, 7, 8, 17, and AT9TSD1 TEs (N=89), respectively (see Methods). For DMR by ∆AB, "GTATTHCM", the most significantly overrepresented motif, matches well with "YAGTATTAY", the motif identified by ChIP-seq (Supplementary Table 1), justifying the approach using DMRs. a: Overrepresented motif. b: Number of sequences matching the motif. c: The p-value of Fisher's Exact Test for enrichment of the motif in the sequences. d: The motif p-value times the number of candidate motifs tested.

Supplementary Table 3.

Divergence between *VANDAL* families in *A. thaliana*

Pair of *VANDAL* families compared		Poisson distance		Number of different repeats in *VANC*
		VANA	*VANC*	
VANDAL6	*VANDAL7*	0.3253	0.5560	3
VANDAL6	*VANDAL8*	0.3740	0.5135	3
VANDAL6	*VANDAL17*	0.3748	0.8727	4
VANDAL6	*VANDAL21-1*	0.4614	0.8609	4~5
VANDAL6	*VANDAL21-2*	0.4644	0.9902	2~4
VANDAL7	*VANDAL8*	0.3648	0.5733	5
VANDAL7	*VANDAL17*	0.4534	0.8991	4
VANDAL7	*VANDAL21-1*	0.5137	0.9163	5~6
VANDAL7	*VANDAL21-2*	0.4472	0.9463	3~5
VANDAL8	*VANDAL17*	0.3450	0.9220	5
VANDAL8	*VANDAL21-1*	0.3546	0.9451	6~7
VANDAL8	*VANDAL21-2*	0.4500	0.9568	4~6
VANDAL17	*VANDAL21-1*	0.3968	0.7703	5~6
VANDAL17	*VANDAL21-2*	0.4929	0.8138	3~5
VANDAL21-1	*VANDAL21-2*	0.2328	0.5404	2~3

Poisson-corrected amino acid genetic distances are averaged from those in copies of complete structure with *VANA* and *VANC*. Number of different repeats in *VANC* coding region was estimated by comparison of multiple copies. From the results in this Table and Supplementary Table 4, separation of these *VANDAL* families were roughly estimated to be in the order of 10 million years, assuming that separation of *A. lyrata* and *A. thaliana* was 5-10 million years ago[21,22].

Supplementary Table 4.

Divergence of *VANDAL* families between *A. thaliana* and *A. lyrata*

	Poisson distance	
	VANA	*VANC*
VANDAL6	0.1440	0.3125
VANDAL7	0.1635	0.3556
VANDAL17	0.1327	0.3229
VANDAL21-2	0.1139	0.3123

Divergence of specific *VANDAL* family between *A. thaliana* and *A. lyrata*. Conditions are as described in Supplementary Table 3. As shown in Supplementary Fig. 14, the distances are several fold lower than those among different *VANDAL* families within *A. thaliana* genome.
