# Supplementary material for: Evolution of sequence-specific anti-silencing systems in Arabidopsis
Source: Nat Commun. 2017 Dec 18;8:2161. doi: 10.1038/s41467-017-02150-7 (PMC5735166; doi:10.1038/s41467-017-02150-7)
Supplement: Supplementary file 3 — Description of Additional Supplementary Files [file 41467_2017_2150_MOESM3_ESM.pdf]

Description of Supplementary Files

File Name: Supplementary Data 1
Description: Effects of VANC21 and VANC6 transgenes on DNA methylation status of TEs longer than 1kb.
